# Sex differences in the C57BL/6 model of Mycobacterium tuberculosis infection

**DOI:** 10.1038/s41598-017-11438-z

**Published:** 2017-09-08

**Authors:** Jannike Dibbern, Lars Eggers, Bianca E. Schneider

**Affiliations:** Division of Coinfection, Priority Area Infections, Research Center Borstel, 23845 Borstel, Germany

## Abstract

Globally, tuberculosis (Tb) notification data show a male-to-female ratio of 1.7 and higher, but the underlying reasons for the male bias remain elusive. Despite the well-known gender bias in human pulmonary Tb, a majority of experimental animal studies either do not separate and analyze data by sex or do not report the sex of their subjects at all. In the present study, we report increased male susceptibility in one of the most commonly used mouse models for Tb, C57BL/6 mice. Our study revealed that disease progression upon aerosol infection with *Mycobacterium tuberculosis* (*Mtb*) was accelerated in males resulting in increased morbidity and mortality compared to females. Elevated *Mtb* loads in males were associated with an early exaggerated pulmonary inflammatory response which likely was detrimental to the host, as reflected by exacerbated pathology and increased mortality. Our data emphasis the urgent need to include and separately analyze both sexes in future animal studies of Tb in order to appreciate the differences in immune responses and disease pathogenesis between males and females.

## Introduction

Tuberculosis (Tb) is the most prevalent bacterial infectious disease in humans. The causative agent, *Mycobacterium tuberculosis* (*Mtb*), is carried by an estimated 2–3 billion people globally and claimed approximately 1.8 million lives in 2015^1^. Tb rates are much higher in men than in women, as reflected by a global male/female ratio of 1.7 for case notifications in 2016^1^. This excess of male pulmonary Tb cases can be observed in adult HIV-negative populations, but not in children and young adolescents^2^. It is widely believed that socioeconomic and cultural factors are responsible for the observed gender bias. Differences in access to health care and the quality of sputum samples are thought to result in under-notification of women and to bias case reporting^2, 3^. Furthermore, confounding factors such as smoking, alcohol or drug use are regarded responsible for higher Tb rates in men. However, several studies now provide evidence that sex differences in Tb are not merely due to differences in case reporting or other confounding factors. Studies in both developing and developed countries reported an excess of Tb cases in males even if confounding factors were taken into account^4–6^. Active case-finding in such surveys could rule out differences in health care access as underlying reason for the observed bias. A recent study on risk factors for Tb in Germany revealed that latently infected household contacts showed a balanced gender distribution but active Tb was more prominent in men^7^. These data suggest an increased risk for disease progression in men.

There is clearly a lack of information on the role of biological sex in Tb. Mouse infection models that reflect the epidemiological observations allow for the analysis of the molecular basis of sex-dependency in Tb disease outcome. Bini and colleagues reported male BALB/c mice to be more susceptible to *Mtb* infection, showing significantly higher bacilli burdens and mortality compared to females and castrated males^8^. The C57BL/6 mouse is the most widely used inbred strain to study experimental Tb. Therefore, it was of major interest to determine potential differences in resistance between the sexes in the C57BL/6 model of Tb. Our study revealed that disease progression upon aerosol *Mtb* infection was accelerated in males resulting in increased morbidity and mortality compared to females. In contrast to BALB/c, elevated *Mtb* loads in male C57BL/6 were associated with exaggerated inflammatory responses and worse pulmonary pathology which probably was detrimental to the host and responsible for the increased mortality.

## Results

### Decreased resistance of male C57BL/6 mice to Mtb infection

**Figure 1:**
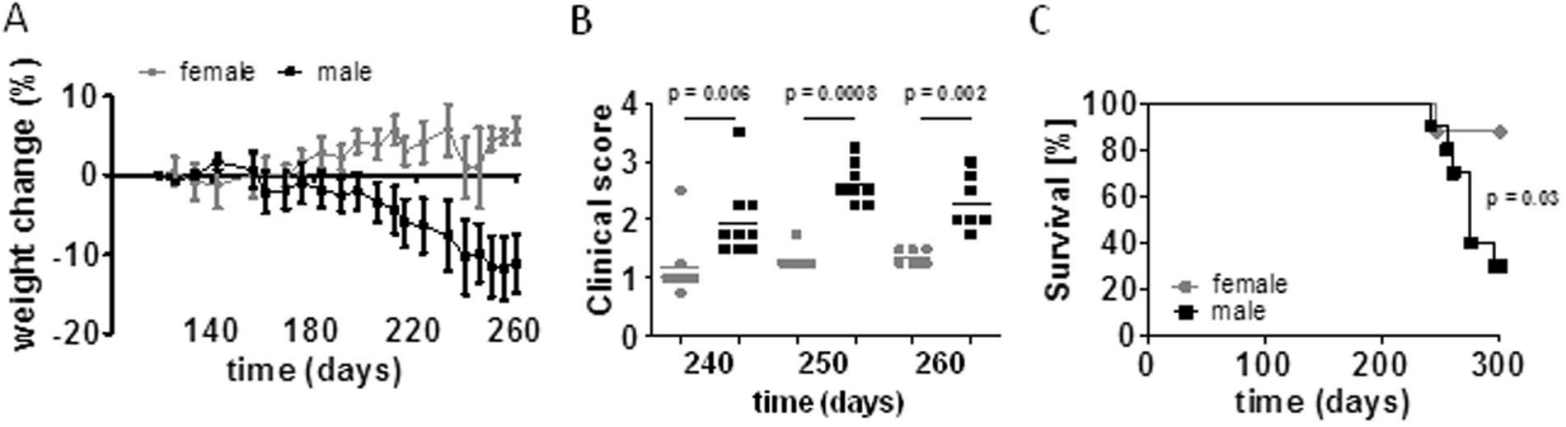
Decreased resistance of male C57BL/6 mice to *Mtb* infection. Male and female C57BL/6 were infected via aerosol with *Mtb* H37Rv and monitored for weight loss (**A**), development of clinical symptoms (**B**), and survival (**C**). Data pooled from two independent experiments are shown as mean ± SD (n = 8–10). Statistical analysis was performed by Mann-Whitney test (clinical score) or log rank test (survival).

Female and male C57BL/6 mice were infected with *Mtb* H37Rv via the aerosol route and monitored for clinical signs of disease as described in materials and methods. Males started to lose weight around day 200 and developed symptoms as reflected by an increase in clinical score (Fig. 1A, B). Consequently, 70% of males succumbed to *Mtb* infection with the remaining animals showing high clinical scores whereas 90% of females survived the observation period of 300 days without overt symptoms (Fig. 1B, C).

**Figure 2:**
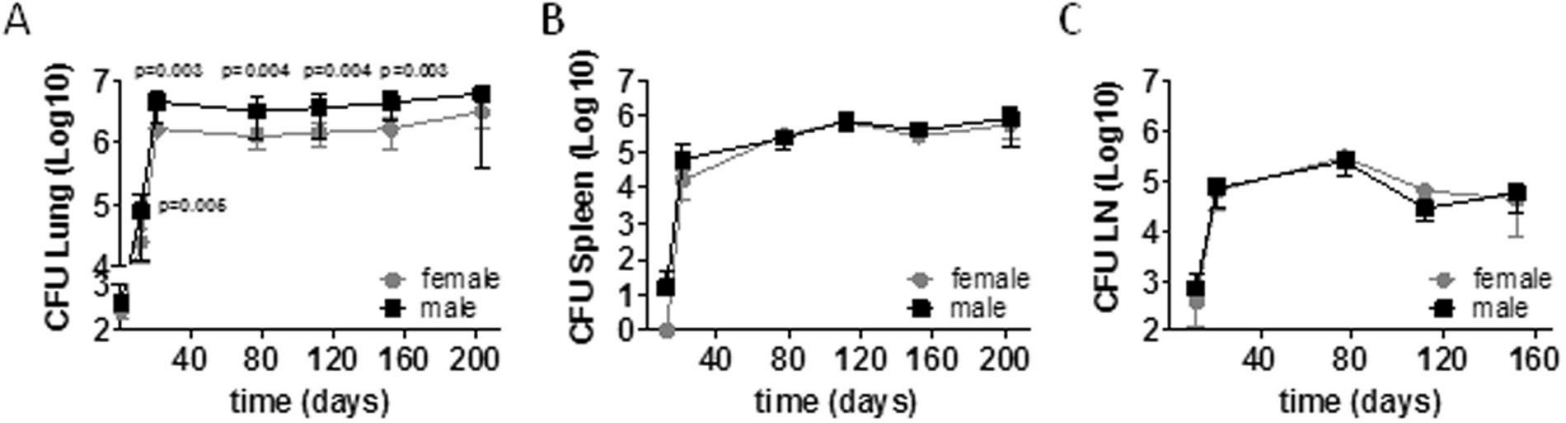
Mycobacterial loads are increased in male lungs. Male and female C57BL/6 were infected via aerosol with *Mtb* H37Rv. At different time points, CFU were determined in organ homogenates. Data pooled from two independent experiments are shown as mean ± SD (n = 8–10). Statistical analysis was performed by Mann-Whitney test.

Mycobacterial loads in male lungs were significantly elevated compared to females but controlled at a steady level (Fig. 2A). We did not find differences in bacterial loads in the spleen and mediastinal lymph node between the sexes (Fig. 2B, C) indicating that bacterial dissemination from the lungs to other organs was not enhanced in males.

In conclusion, we found increased mycobacterial loads, accelerated disease progression and premature death upon aerosol *Mtb* infection in C57BL/6 males compared to females.

### Pulmonary pathology develops differently in males and females

**Figure 3:**
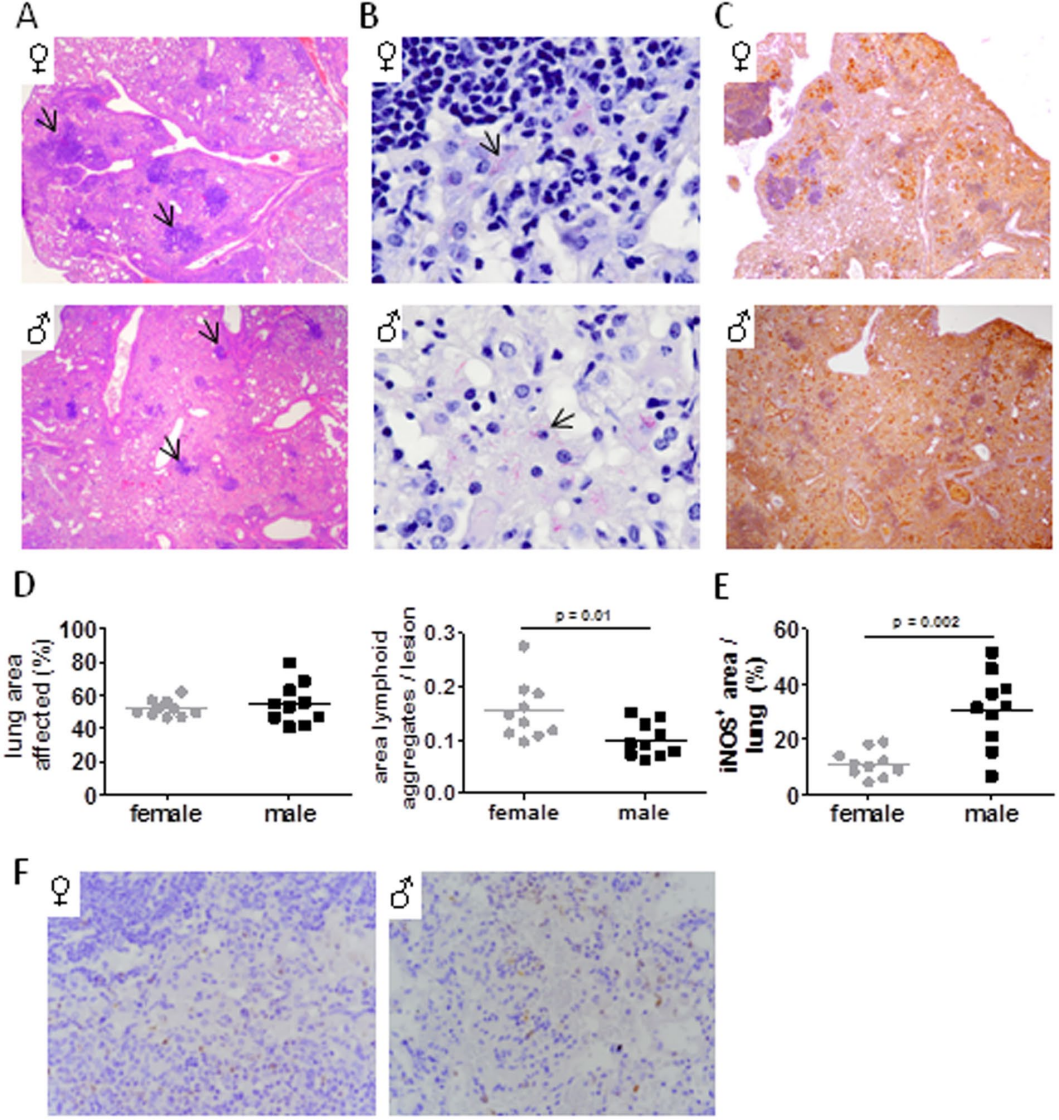
Histological characteristics of *Mtb* infected lungs. Male and female C57BL/6 were infected via aerosol with *Mtb* H37Rv and histologically evaluated after 152 days. (**A**) Granulomatous lesions in H&E stained lung sections (arrows: lymphoid aggregates). (**B**) Acid fast bacilli (arrows). (**C**) Immunohistochemical staining of iNOS. Representative micrographs from one mouse out of 10 mice per group are shown (original magnification × 40 in **A** and **C**; × 400 in **B**). (**D**) Quantitative assessment of total lung area affected by lesions (left graph) and the ratio of lymphoid aggregates to lesion area (right graph). (**E**) Quantitative evaluation of iNOS expression shown in (**C**. **F**) Immunohistochemical staining of neutrophils (Ly6B.2). Representative micrographs from one mouse out of 10 mice per group are shown (original magnification × 100).

Histopathological examination of lungs five months after *Mtb* infection revealed progressive pathology with striking differences in the quality of the granulomatous lesions between the sexes. We observed typical lesions consisting of characteristic foamy macrophages surrounding a lymphocytic core structure in females (Fig. 3A, arrows). In contrast, these lymphoid aggregates appeared much smaller in size in males. Quantitative analysis indeed revealed that the overall lung area covered by lesions was comparable between the sexes, but the ratio of lymphocytic aggregate to lesion area was significantly reduced in males (Fig. 3D). Instead, surrounding myeloid infiltrates dominated in male lungs and covered greater regions compared to females, suggesting differences in immune cell recruitment and spatial organization. Mycobacteria were mainly associated with foamy macrophages in both sexes but observed in greater numbers in males (Fig. 3B, arrows), confirming CFU data. Increased mycobacterial burden in males prompted us to analyse iNOS expression which to our surprise was much more abundant and ubiquitously distributed in the male lung tissue while its expression was more discrete and localized to areas surrounding lymphoid infiltrates in females (Fig. 3C). Quantitative analysis of histological sections confirmed that iNOS expression was significantly increased in male compared to female lungs (Fig. 3E).These data indicate that in males, the immune response is dysregulated and generates an inflammatory response that leads to worse pulmonary pathology that is detrimental to the host, as reflected by increased mortality.

### Pulmonary inflammatory responses to Mtb are enhanced in males

**Figure 4:**
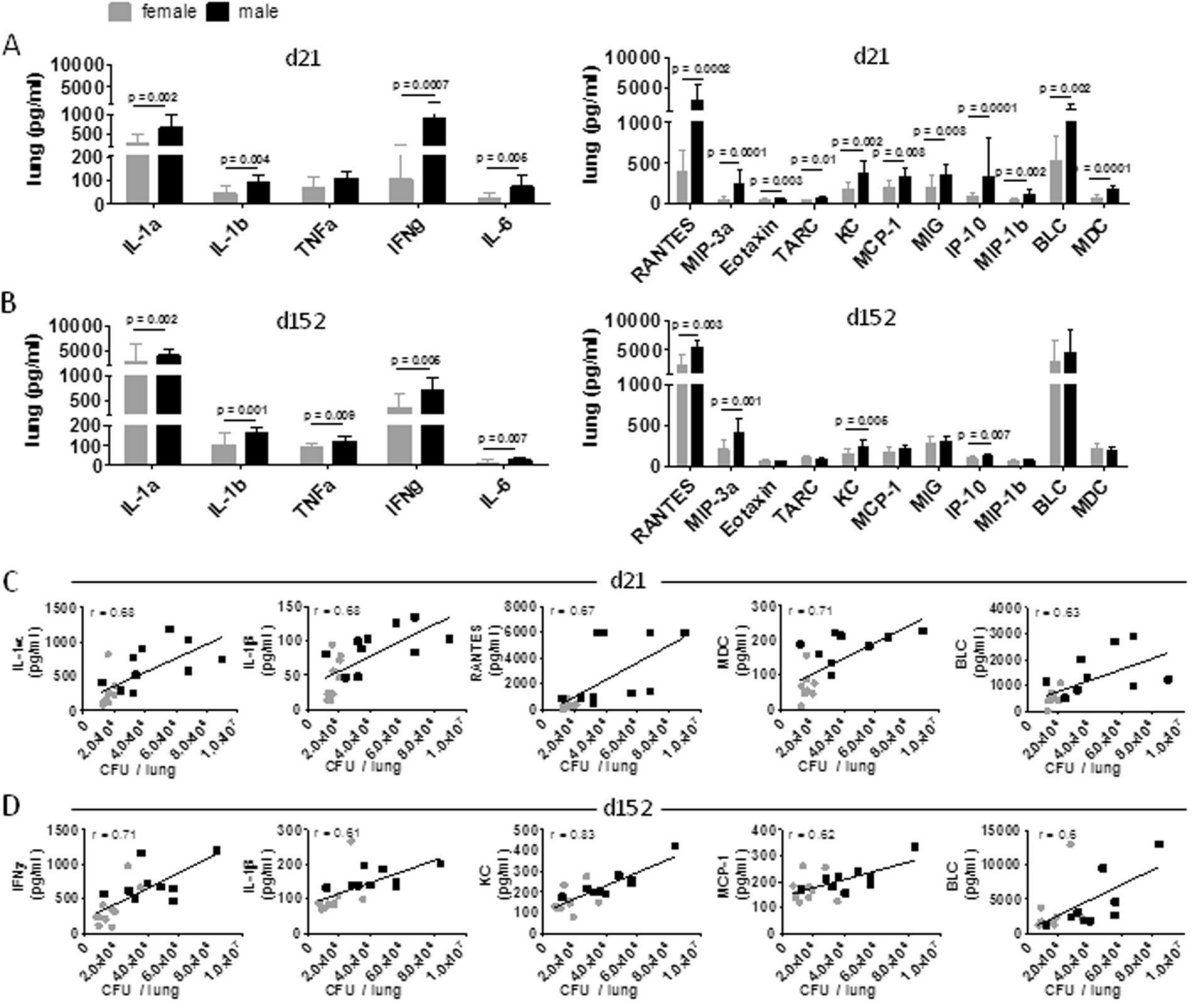
Increased inflammatory responses upon *Mtb* infection in male lungs. Cytokine and chemokine levels were measured in lung lysates of female and male C57BL/6 mice on day 21 (**A**) and 152 (**B**) after *Mtb* H37Rv aerosol infection. Data pooled from two independent experiments are shown as mean ± SD (n = 9–10). Statistical analysis was performed by Mann-Whitney test. Correlations between cyto- or chemokines plotted versus bacterial burden in in lungs for day 21 (**C**) or d152 (**D**) in males and females. Each dot represents an individual sample (n = 9–10; females gray; males black).

In order to identify immune differences in female and male lungs that might explain differences in pathology observed we next analyzed the production of a number of relevant cytokines and chemokines early and late during *Mtb* infection. Among those tested the pro-inflammatory mediators IL1α, IL1β, IFNγ and IL-6 and numerous chemokines were substantially elevated in male lungs as early as 21 days post infection (Fig. 4A). All cytokines, RANTES, MIP-3α, KC and IP-10 and in addition TNFα were still elevated in males after 152 days (Fig. 4B), indicating sustained inflammation. Correlation analysis including all mice revealed that certain cyto- and chemokines were positively correlated with bacterial burden in lungs on day 21 and 152 of *Mtb* infection, respectively (Fig. 4C and D). When separating individual mice by sex it became obvious that those mice that showed high CFU and high cyto- or chemokine levels were predominantly males. Since the strongest positive correlation in males was observed for KC in late infection, we stained lung sections with the anti-neutrophil antibody 7/4 (recognizing Ly-6B.2) to see whether increased neutrophil recruitment was apparent in males. However, we only found few neutrophils in both sexes and no apparent difference or increase in male lungs (Fig. 3F).

Our results suggest that differences in early cytokine and chemokine signaling during pulmonary Tb contribute to the magnitude and quality of the granulomatous response, which participate in differences in disease development and progression between males and females.

## Discussion

Many studies in humans and experimental animals have established clear links between sex-specific factors and the differential susceptibility of males and females to a number of infectious and noninfectious diseases^9, 10^. Generally, females mount higher innate and adaptive immune responses than males, which enables faster clearance of pathogens but increases susceptibility to inflammatory and autoimmune diseases^10^. A few years ago the NIH announced new policies to ensure that preclinical research funded by the NIH considers both males and females^11^ however many experimental studies continue to neglect sex-based considerations and analyses of data by sex. Likewise, despite the well-known gender bias in human pulmonary Tb, a majority of experimental animal studies either do not separate and analyze data by sex or do not report the sex of their subjects at all. Importantly, sex differences in mycobacterial infections are not only observed in humans but also in wildlife. *M. bovis* infection is commonly reported in deer where males seem to be more affected than females^12, 13^. Moreover, an important reservoir and source of bovine tuberculosis in cattle is the European badger, where females are more resilient to established *M. bovis* infection than male badgers and present with longer survival times following the detection of bacterial excretion^14^. Therefore, there is an urgent need to investigate the molecular basis of sex-dependency in Tb disease outcome. We show here for the first time that the widely used C57BL/6 model of experimental Tb reflects the epidemiological findings of increased male susceptibility. Like in BALB/c^8^, aerosol *Mtb* infection resulted in increased morbidity and mortality in C57BL/6 males compared to females, indicating that increased male susceptibility is independent of the genetic background. In clear contrast to BALB/c, male C57BL/6 mice responded with augmented and sustained pulmonary inflammation to *Mtb* infection. Profound differences between the sexes were already observed after three weeks with overproduction of hallmark pro-inflammatory cytokines IL1α, IL1β and IFNγ and a broad range of chemokines being indicative of excessive inflammation in response to *Mtb* infection in males. Despite a robust pro-inflammatory immune response, mycobacterial loads in male lungs were significantly increased, indicating that *Mtb* induces a sustained non-protective inflammatory reaction in the male lung that is detrimental to the host, as reflected by increased mortality. Our results are in line with the overall conception that cytokines which are critically required for resistance to *Mtb* infection become potentially destructive when produced uncontrolled. For instance, IL1β production needs to be controlled at the inflammasome level in order to prevent detrimental innate inflammatory responses during *Mtb* infection^15^ and a polymorphism in the human *IL1B* promoter region associated with increased production of IL1β is associated with active Tb, severity of disease and poor treatment outcome^16^. Recently, Sakai and colleagues elegantly demonstrated that increasing pulmonary IFNγ production exacerbated *Mtb* infection and that negative regulation of IFNγ production is required to prevent lethal immune-mediated pathology^17^.

In line with elevated pro-inflammatory mediators was the increased pulmonary pathology characterized by reduced lymphoid aggregates but large areas of myeloid cell infiltrates in male lungs. Despite increased levels of KC in males, neutrophils were hardly detected in lungs of both sexes and thus were unlikely to contribute to pathology in males. In contrast to more susceptible mouse models such as C3H, DBA or I/St, the C57BL/6 mouse model of Tb is not associated with neutrophil accumulation and neutrophil-driven pathology^18–21^ and neutrophil depletion in C57BL/6 mice did not increase mycobacterial loads in different studies^22, 23^. Increased IFNγ in males might explain why despite the high levels of KC neutrophils were not found to accumulate in male lung tissue. Nandi and Behar have demonstrated that IFNγ impairs neutrophil survival and inhibits pathogenic neutrophil accumulation in the infected lung^24^ presumably by suppressing the expression of E- and P-selectin on endothelial cells which are important for neutrophil trafficking into inflamed tissue^25, 26^ and by the induction of iNOS expression. NO prevents neutrophil recruitment^15, 27^ while its absence results in increased neutrophil recruitment and tissue necrosis at the site of *Mtb* infection^15, 27–30^. Thus, increased IFNγ together with IL1 likely induced the strong and ubiquitous iNOS expression in male lung tissue and thereby prevented the accumulation of neutrophils. Moreover, the ratio of local to systemic chemokine concentrations seems to be critical for local neutrophil recruitment^31^. We did not determine systemic cyto- or chemokine levels and therefore can only speculate that systemic levels of KC might even exceed those of local concentrations determined in infected lung tissue.

Instead of neutrophils, the most prominent myeloid cell in *Mtb* infected C57BL/6 lungs is the macrophage^20^ and in particular foamy macrophages are key players in sustaining persistent bacteria and tissue pathology^32^. Reports by the group of Sher and Ramakrishnan demonstrated that the recruitment of a permissive monocyte/macrophage population in a CCL2/CCR2 dependent manner is detrimental in *Mtb* infection^33, 34^. We believe that more permissive macrophages are recruited to male compared to female lungs and less lymphocytes as reflected by smaller lymphoid cores, and ultimately promote disease progression and premature death.

It has become increasingly clear that early events in *Mtb* infection are of major importance in dictating clinical outcome. Data from nonhuman primate models suggest that between 3–6 weeks post infection, one can predict whether the animal will progress to active Tb or remain latent until 6–9 month later^35^. The numbers of initial granulomas and increasing inflammation in the first 6 weeks were associated with development of active Tb. Moreover, early greater IFNγ production was observed among macaques that would later develop active Tb than for those that developed latent infection^36^. Likewise, in a panel of genetically heterogeneous mice, Tb progression correlated with increased lung expression of inflammation-related factors^37^. The identification of biomarkers or transcriptional signatures that prospectively predict progression of *Mtb* infection to active disease is a great area of research. However, lung or BAL samples are not available for routine analysis and thus cannot be used to predict disease progression. Instead whole blood transcriptional profiling offers great prognostic potential and several groups have identified transcripts that are associated with active Tb in humans^38–40^. Together the different studies suggest an inflammatory manifestation of Tb and a predictive signature identified by Zak *et al*. suggests that peripheral activation of an IFN-inducible transcriptional signature which was also associated with active Tb in other studies precedes the onset of active disease^41^. Peripheral responses might differ from those observed locally at the site of infection and differences in *Mtb* specific T cell responses between BAL fluid and PBMCs were demonstrated among active Tb patients^42, 43^. Nevertheless, human blood transcriptional signatures in active Tb are supported by data from experimental *Mtb* infection models which consistently show that excess inflammation in the lung is associated with progressive Tb. In a macaque model, Gideon and colleagues could demonstrate that early blood transcript activity was highly correlated with lung inflammation^44^, indicating that indeed peripheral responses reflect events at the site of infection. Moreover, analysis of preinfection signatures of macaques revealed that IFN signatures could influence eventual clinical outcomes even before infection, reflecting the observations in humans. In order to better complement epidemiological studies it will be important to analyse blood levels of immune mediators in our mouse model of experimental *Mtb* infection in the future. This is of particular interest in light of preinfection signatures predictive for the risk to develop active Tb disease which may differ between the sexes. Inherent functional differences in innate and adaptive immune mechanisms involved in early host-pathogen interaction likely influence the overall outcome of *Mtb* infection. It is well known that innate recognition of pathogens and the induction of inflammatory immune responses differ between the sexes, one reason being the influence of sex steroid hormones^10, 45^. Alveolar macrophages are the first cells that encounter *Mtb* in the host lung and initiate a local inflammatory immune response^18^. In addition, macrophages, together with DCs, serve as antigen-presenting cells that initiate and regulate effector T-cell responses. Scotland and colleagues reported on fundamental sex differences in resident immune cell phenotype in the peritoneal cavity of mice which was reflected by greater TLR expression and enhanced phagocytosis and bacterial killing in females compared to males^46^. On the other hand, macrophage-derived cytokine production was diminished in females because of proportionally more immunomodulatory CD4^+^ T-cells. Likewise, Yang and colleagues observed superior bacterial killing by female alveolar macrophages^47^ accompanied by diminished lung inflammation and better survival. Thus, while females show superior anti-bacterial killing over males, they seem to counterbalance excess inflammation which results in better survival. We observed increased mycobacterial loads in male lungs as early as 12 days after infection. Thus, an important question to answer in the future is whether mycobacterial killing is impaired in alveolar macrophages of males. Overproduction of pro-inflammatory mediators secondary to elevated bacterial loads may perpetuate exaggerated inflammatory responses leading to inappropriate immune cell trafficking and activation in the male lung which ultimately leads to increased mortality of males compared to females. Alternatively, the ability to regulate inflammatory responses induced by *Mtb* might be impaired in males.

To the best of our knowledge, this is the first report showing sex differences in the commonly used C57BL/6 model of aerosol *Mtb* infection. Although the increased susceptibility of males only becomes apparent during late-stage infection with regard to clinical score and survival, this phenotype clearly is associated with an augmented early inflammatory immune response which presumably contributes significantly to the accelerated disease development and progression in males. Therefore, our data emphasis the need to include and separately analyze both sexes in future animal studies of Tb in order to appreciate the differences in immune responses and disease pathogenesis between males and females. A comprehensive understanding of the sex disparity in Tb will help to develop prognostic tools or host-directed therapies and may be important when evaluating the effectiveness of vaccines and other immunological interventions.

## Methods

### Ethics Statement

Animal experiments were in accordance with the German Animal Protection Law and approved by the Ethics Committee for Animal Experiments of the Ministry of Energy, Agriculture, Environment, and Rural Areas of the State of Schleswig-Holstein under the license 103–9/14.

### Mice, bacterial infection and colony forming units (CFU)

C57BL/6 mice were bred under specific-pathogen-free conditions at the Research Center Borstel. Female and male C57BL/6 mice aged between 8–12 weeks were used and maintained under specific barrier conditions in BSL 3 facilities.

*Mtb* H37Rv was grown in Middlebrook 7H9 broth (BD Biosciences) supplemented with 10% v/v OADC (Oleic acid, Albumin, Dextrose, Catalase) enrichment medium (BD Bioscience). Bacterial aliquots were frozen at −80 °C. Viable cell counts in thawed aliquots were determined by plating serial dilutions onto Middlebrook 7H10 agar plates supplemented with 10% v/v heat-inactivated bovine serum followed by incubation at 37 °C.

For infection of experimental animals, *Mtb* stocks were diluted in sterile distilled water at a concentration providing an uptake of 200 viable bacilli per lung. Infection was performed via the respiratory route by using an aerosol chamber as described previously^48^. The inoculum size was quantified 24 h after infection by determining CFU in the lungs of infected mice. CFU in lung, mediastinal lymph nodes and spleen were evaluated at different time points after aerosol infection by mechanical disruption of the organs in 0.05% v/v Tween 20 in PBS containing a proteinase inhibitor cocktail (Roche) prepared according to the manufacturer’s instructions. Tenfold serial dilutions of organ homogenates in sterile water/1% v/v Tween 80/1% w/v albumin were plated onto Middlebrook 7H11 agar plates supplemented with 10% v/v heat-inactivated bovine serum and incubated at 37 °C for 3–4 weeks.

### Evaluation of disease

A clinical score was used to indicate severity of disease progression. Animals were scored in terms of general behavior, activity, feeding habits, and weight gain or loss. Animals with severe symptoms (reaching a clinical score of ≥ 3.5) were euthanized to avoid unnecessary suffering, and the time point that followed was denoted the time of death.

### Multiplex cytokine assay

The concentrations of various cytokines and chemokines in lung homogenates were determined by LEGENDplex^TM^ (Mouse Inflammation panel and Mouse Proinflammatory Chemokine Panel, BioLegend) according to the manufacturer’s protocol.

### Histology

Superior lung lobes from infected mice were fixed with 4% w/v paraformaldehyde for 24 h, embedded in paraffin, and sectioned (4 µm). Sections were stained with hematoxylin and eosin (H&E), carbol fuchsin (Merck) followed by decolorization with acid-alcohol to visualize mycobacteria in the lungs. iNOS or neutrophils were detected by a polyclonal rabbit anti inducible nitric oxid synthase (iNOS; Merck Millipore) or monoclonal rat anti neutrophils (clone 7/4; Cedarlane), respectively, followed by secondary antibody (biotinylated goat anti rabbit; Dianova), amplification (avidin-HRP) and color reaction (DAB solution; Vectastain). Slides were imaged with a BX41 light microscope and cell^B software. The quantitative analysis of lesion size and lymphoid aggregates was conducted by determination of whole lung area (5–8 images per mouse) as well as the lesion area of H&E stained slides with cell^B. The same images were analyzed by a second person to determine the area of lymphocytic aggregates per lung using the software Fiji ImageJ (freeware). The analyses were performed in a blinded manner. Quantification of iNOS expression was analyzed by ImageJ (1–2 representative images per mouse). Hematoxylin and DAB color were deconvolved (pre-existing plug-in by ImageJ) and the threshold was set for the DAB component to exclude unspecific signals. Lung area of the representative images was determined and the proportion of iNOS signal was calculated. iNOS signals of blood vessels were excluded.

### Statistical Analysis

Statistical analysis was performed by Mann-Whitney test or by log rank test as described in the figure legends. Correlation between variables was determined by calculating Pearson’s coefficient using a 2-tailed analysis. A correlation was taken into account as of r ≥ 0.60 (defined as strong correlation). Significance was defined as P ≤ 0.05. All data were analysed using GraphPad Prism 5 (GraphPad Software, Inc.).
