# Author Correction: Sex differences in the C57BL/6 model of Mycobacterium tuberculosis infection

**DOI:** 10.1038/s41598-018-24598-3

**Published:** 2018-04-17

**Authors:** Jannike Dibbern, Lars Eggers, Bianca E. Schneider

**Affiliations:** Division of Coinfection, Priority Area Infections, Research Center Borstel, 23845 Borstel, Germany

Correction to: *Scientific Reports* 10.1038/s41598-017-11438-z, published online 08 September 2017

The Acknowledgements section in this Article is incomplete.

“We thank Doreen Beyer for culturing of Mtb H37Rv, Imke Storm for technical assistance, Johanna Volz for helpful discussions regarding immunohistochemistry and Jochen Behrends and Hannelore Lotter for critical discussions and helpful comments on the manuscript. This work was supported by in-house funding from the Research Center Borstel.”

should read:

“We thank Doreen Beyer for culturing of *Mtb* H37Rv, Imke Storm for technical assistance, Johanna Volz for helpful discussions regarding immunohistochemistry and Jochen Behrends and Hannelore Lotter for critical discussions and helpful comments on the manuscript. This work was supported by in-house funding from the Research Center Borstel. The publication of this article was funded by the Open Access Fund of the Leibniz Association.”

